# Astrocyte-Dependent Slow Inward Currents (SICs) Participate in Neuromodulatory Mechanisms in the Pedunculopontine Nucleus (PPN)

**DOI:** 10.3389/fncel.2017.00016

**Published:** 2017-02-01

**Authors:** Adrienn Kovács, Balázs Pál

**Affiliations:** Department of Physiology, Faculty of Medicine, University of DebrecenDebrecen, Hungary

**Keywords:** pedunculopontine nucleus, slow inward current, optogenetics, astrocyte, neuromodulation

## Abstract

Slow inward currents (SICs) are known as excitatory events of neurons caused by astrocytic glutamate release and consequential activation of neuronal extrasynaptic NMDA receptors. In the present article we investigate the role of these astrocyte-dependent excitatory events on a cholinergic nucleus of the reticular activating system (RAS), the pedunculopontine nucleus (PPN). It is well known about this and other elements of the RAS, that they do not only give rise to neuromodulatory innervation of several areas, but also targets neuromodulatory actions from other members of the RAS or factors providing the homeostatic drive for sleep. Using slice electrophysiology, optogenetics and morphological reconstruction, we revealed that SICs are present in a population of PPN neurons. The frequency of SICs recorded on PPN neurons was higher when the soma of the given neuron was close to an astrocytic soma. SICs do not appear simultaneously on neighboring neurons, thus it is unlikely that they synchronize neuronal activity in this structure. Occurrence of SICs is regulated by cannabinoid, muscarinic and serotonergic neuromodulatory mechanisms. In most cases, SICs occurred independently from tonic neuronal currents. SICs were affected by different neuromodulatory agents in a rather uniform way: if control SIC activity was low, the applied drugs increased it, but if SIC activity was increased in control, the same drugs lowered it. SICs of PPN neurons possibly represent a mechanism which elicits network-independent spikes on certain PPN neurons; forming an alternative, astrocyte-dependent pathway of neuromodulatory mechanisms.

## Introduction

Slow inward currents (SICs) are neuronal events with significantly slower kinetics than the spontaneous or miniature excitatory postsynaptic currents (sEPSCs). The amplitude of SICs is generally larger, but there might be overlaps in the amplitude ranges with EPSCs. As SICs are slower, rise and decay times are even better tools to distinguish the two types of events; the decay phase of the SICs can be well fitted with a single exponential function, whereas the declining phase of EPSCs is ideally fitted by double exponential function (Fellin et al., 2004; Shigetomi et al., 2008; Bardoni et al., 2010; Reyes-Haro et al., 2010; see Pál, 2015). SICs are thought to be consequences of activation of extrasynaptic, GluN2B-containing NMDA receptors (Angulo et al., 2004; Fellin et al., 2004; Kozlov et al., 2006; D’Ascenzo et al., 2007; Nie et al., 2010; Pirttimaki et al., 2011; Pirttimaki and Parri, 2012). SICs can also be distinguished from EPSCs based on their origin. These events can be elicited by astrocytic activation and consequential astrocytic glutamate release; thus substances activating astrocytes are potentially capable of eliciting SICs, whereas inhibition of astrocytes prevents generation of SICs on neurons (Perea and Araque, 2005; D’Ascenzo et al., 2007; Bardoni et al., 2010; Pirttimaki et al., 2011; Chen et al., 2012; Perea et al., 2014; see Pál, 2015). SICs were detected on several areas of the central nervous system (CNS; e.g., hippocampus, olfactory bulb, visual cortex, thalamus, nucleus accumbens, medial nucleus of the trapezoid body and the spinal cord), thus seem to be a general feature of it (e.g., Carmignoto and Fellin, 2006; Kozlov et al., 2006; D’Ascenzo et al., 2007; Nie et al., 2010; Reyes-Haro et al., 2010; Chen et al., 2012; see Pál, 2015).

The physiological significance of SICs is possibly the synchronization of neurons located close to each other, following extensive stimulation of a strong input to the structure in question (Angulo et al., 2004; Fellin et al., 2004; D’Ascenzo et al., 2007; Pirttimaki et al., 2011). However, this observation might not be applicable for the brainstem, as synchronization by SICs rarely occurred in the medial nucleus of the trapezoid body (Reyes-Haro et al., 2010). Large SICs of cortical areas are likely have pathophysiological significance, as they are capable of synchronizing neuronal activity and facilitating seizure generation (Gao and Goldman-Rakic, 2006; Wetherington et al., 2008), and have a contribution to the pathophysiology of stroke and cerebral edema (Dong et al., 2013; Lauderdale et al., 2015).

The pedunculopontine nucleus (PPN) is a cholinergic nucleus in the brainstem, having a significant role in modulation of sleep and wakefulness, as well as in regulation of movement (see e.g., Steriade et al., 1990; Garcia-Rill, 1991; Reese et al., 1995). It is composed of cholinergic and non-cholinergic (GABAergic, glutamatergic) neurons, which display different activity patterns correlated with cortical activity (Mena-Segovia et al., 2008; Ros et al., 2010; Petzold et al., 2015). In general, cortical slow wave activity was coupled with the highly correlated activity of PPN neuronal populations, and decorrelated activity was seen during cortical desynchronization (Petzold et al., 2015). PPN neurons are targets of several neuromodulatory actions. Injections of cannabinoid or cholinergic agonists to the PPN are capable of changing sleep-wake states of the experimental animals, and serotonergic and orexinergic actions on the global brain states are also well known (Bjorvatn and Ursin, 1998; Kinney et al., 1998; Murillo-Rodríguez et al., 2008; Matulewicz et al., 2010; Mieda et al., 2011; Valencia et al., 2014).

Special patterns of tonic activation and inhibition of PPN neurons were identified behind the actions depicted above. The cholinergic agonist carbachol as well as serotonin can depolarize or hyperpolarize neurons in this nucleus (Leonard and Llinás, 1994; Ye et al., 2010; Grace et al., 2012). Similarly, endocannabinoids also depolarized or hyperpolarized PPN neurons by eliciting tonic currents. We previously demonstrated that the tonic currents on PPN neurons develop via activation of astrocytes (Kőszeghy et al., 2015; Kovács et al., 2015, 2017). Astrocytes, in turn, release glutamate, which depolarize or hyperpolarize neurons in a tonic way via activation of different sets of metabotropic glutamate receptors. However, these tonic currents might not be the only form of astrocyte-neuron communication in the PPN.

Our aim was to determine whether SICs participate in neuromodulatory mechanisms and/or neural synchronization within the PPN. We found that SICs can be clearly separated from EPSCs on a portion of cholinergic and GABAergic PPN neurons and they are the consequences of astrocytic glutamate release and activation of extrasynaptic NMDA receptors. SICs cause transient depolarizing periods and single action potentials or short trains of action potentials, but they do not seem to represent a local mechanism for synchronization of neighboring neurons in the PPN. Different neuromodulatory mechanisms act on SIC frequency in an overlapping way: independently from the applied agonist, initially low SIC frequency is increased, while originally high SIC activity is repressed.

## Materials and Methods

### Solutions, Chemicals

Artificial cerebrospinal fluid (aCSF) was used for slice electrophysiology experiments with the following composition (in mM): NaCl, 120; KCl, 2.5; NaHCO_3_, 26; glucose, 10; NaH_2_PO_4_, 1.25; myo-inositol, 3; ascorbic acid, 0.5; sodium-pyruvate, 2; CaCl_2_, 2; MgCl_2_, 1; osmolarity, 325 mOsm/l; pH 7.2. Preparation was performed in low Na^+^ aCSF. In this solution, 95 mM NaCl was replaced by glycerol (60 mM) and sucrose (130 mM). In several patch clamp experiments, a nominally magnesium-free aCSF was used, as MgCl_2_ was not administered to the solution. All chemicals were purchased from Sigma (St. Louis, MO, USA), unless stated otherwise.

### Animals, Preparation

We conducted all animal experiments in accordance with the appropriate international (EU Directive 2010/63/EU for animal experiments) and Hungarian laws and institutional guidelines on the care of research animals. Our experimental protocols were approved by the Committee of Animal Research of the University of Debrecen (5/2015/DEMÁB). The homozygous floxed-stop- tdTomato (B6;129S6-Gt(ROSA)26Sor^tm9(CAG-tdTomato)Hze/^J; Jax mice accession number: 007905), -channelrhodopsin-2 (ChR2) (B6;129S-Gt(ROSA)26Sor^tm32(CAG-COP4*H134R/EYFP)Hze/^J; Jax number: 012569), glutamate decarboxylase type 2 (GAD2)-cre (Gad2^tm2(cre)Zjh/^J; Jax number: 010802), choline acetyltransferase (ChAT)-cre (B6;129S6-Chat^tm2(cre)Lowl/^J; Jax number: 006410) and glial fibrillary acidic protein (GFAP)-cre (B6.Cg-Tg(Gfap-cre)73.12Mvs/J; Jax number: 012886) strains with C57BL6/J background originated from Jackson Laboratories (Bar Harbor, ME, USA) and were crossed in the animal facility of the University of Debrecen, Department of Physiology, following the recommendations of Jackson Laboratories (Kőszeghy et al., 2015; Kovács et al., 2015, 2017). Eight to 16 days old offsprings heterozygous both for the cre driver and the floxed-stop genes, expressing tdTomato fluorescent protein in a GFAP-, ChAT- or GAD2- dependent way (*n* = 18, 17 and 10, respectively), as well as animals expressing ChR2 in a GFAP-dependent way, from both sexes (*n* = 17), were used for the slice electrophysiology experiments. Although the GFAP-cre line is not fully specific for astrocytes, but neurons in certain hippocampal (and, more rarely, cerebellar or brainstem) regions also have Cre recombinase activity, expression appears to be specific to astrocytes within the PPN (Garcia et al., 2004).

Coronal midbrain slices (with 200 μm thickness) were prepared in low Na^+^ aCSF (cca. 0 to −2°C) with a Microm HM 650V vibratome (Microm International GmbH, Walldorf, Germany). The slices were incubated in normal aCSF (nACSF) for 1 h on 37°C prior to starting the experiment.

### Electrophysiology

The resistance of the patch pipettes was 5 MΩ, and the composition of the internal solution was the following (in mM): K-gluconate, 120; NaCl, 5; 4-(2-hydroxyethyl)-1-piperazineethanesulfonic acid (HEPES), 10; Na2- phosphocreatinine, 10; EGTA, 2; CaCl_2_, 0.1; Mg-ATP, 5; Na_3_-GTP, 0.3; biocytin, 8; osmolarity, 285–290 mOsm/l; pH 7.3;. Whole-cell patch-clamp experiments were conducted at room temperature with Axopatch 200A amplifiers (Molecular Devices, Union City, CA, USA). Clampex 10.0 software (Molecular Devices, Union City, CA, USA) was used for data acquisition, while data analysis was performed by Clampfit 10.0 (Molecular Devices) and MiniAnalysis (Synaptosoft, Decatur, GA, USA) softwares. The amplitude of SICs was defined as the difference of the average of datapoints from a 20-ms-long trace prior to the onset of the event and the peak of the event. Zero to one hundred percent rise and 10%–90% decay time was taken into account, and the decay of the EPSCs were fitted with double and single exponential fits, whereas only single exponential fit was used in case of SICs. The area of SICs was calculated with the numerical integration of datapoints from the onset of the event to that point of the trace where the current value of the onset was recorded again.

For recording SICs, tonic currents, EPSCs and IPSCs, voltage-clamp traces were recorded at a holding potential of −60 mV before and after drug application or optogenetic manipulation (10 min in each condition). Only stable recordings with minimal leak currents and histograms with a single peak were used for analysis. The series resistance was monitored during recordings and only recordings with series resistance below 30 MΩ, with less than 10% change were accepted. Numerical values of the tonic currents were calculated from histograms of 1-min long current traces and the peak value of the histogram (i.e., the largest population of data points from the trace) was considered as the magnitude of the tonic current. Ten minutes long recordings were performed under control conditions to measure the spontaneous fluctuations of the holding current between the first and last minute of the recording.

To assess whether SICs can elicit action potential firing on neurons, a representative SIC (89 pA amplitude, 18.8 ms rise time, 80 ms decay tau; recorded on −60 mV holding potential from a PPN neuron) was used as an input signal in current-clamp mode, administered at −60 mV membrane potential of the given neuron. Paired recordings were performed in voltage-clamp mode. Somata within 20 μm from each other were patched simultaneously and currents were recorded on them at −60 mV holding potential. Pairs of SICs occurring in a time window of ±2 s were considered coincident.

Optogenetic activation of GFAP-ChR2 samples was performed by using continuous illumination with external LED (470 nm; Thorlabs, Newton, NJ, USA). Astrocyte activation by optogenetic stimulation in GFAP-ChR2 mice was previously confirmed by calcium imaging and patch-clamp experiments (see Kovács et al., 2017). Flash photolysis of MNI-caged glutamate (100 μM; Tocris Cookson Ltd., Bristol, UK) was achieved by Rapp flash lamp JML-C2 (Rapp OptoElectronic GmbH, Wedel, Germany); the entire visual field was illuminated during uncaging.

Visualization of genetically encoded fluorescent markers (tdTomato, EYFP) was achieved by a fluorescent imaging system (Till Photonics GmbH, Gräfeling, Germany) containing a xenon bulb-based Polychrome V light source, a CCD camera (SensiCam, PCO AG, Kelheim, Germany), an imaging control unit (ICU) and the Till Vision software (version 4.0.1.3).

### Pharmacology

In some experiments, 1 μM tetrodotoxin (TTX; Alomone Laboratories, Jerusalem, Israel) was administered to eliminate action potential generation in the preparation. The GluN2B-specific NMDA receptor blocker ifenprodil was used in 5 μM, whereas the general NMDA receptor antagonist D(-)-2-Amino-5-phosphonopentanoic acid (D-AP5) was administered in 50 μM concentration (Tocris Cookson Ltd., Bristol, UK). The glutamate transporter inhibitor DL-threo-β-Benzyloxyaspartic acid (DL-TBOA; Tocris Cookson Ltd., Bristol, UK) was applied in 10 μM concentration. In certain experiments, slices were treated with 1 μM thapsigargin for 45 min to inhibit astrocytic calcium wave activity (Kőszeghy et al., 2015)., WIN55,212-2 (1 μM) was used as CB1 receptor agonist, and the muscarinic agonist carbamylcholine chloride (carbachol) was administered in 50 μM. Serotonin was administered in 10 μM. At the end of some experiments, slices were washed with 10 μM 2,3-dihydroxy-6-nitro-7-sulfamoyl-benzo[f]quinoxaline-2,3-dione (NBQX), 50 μM D-AP5, 1 μM strychnine and 10 μM bicuculline (Tocris Cookson Ltd., Bristol, UK) in order to block fast synaptic neurotransmission.

### Morphological Analysis of the Recorded Neurons and Immunohistochemistry

Patched neurons were labeled with biocytin and samples were fixed (4% paraformaldehyde in 0.1 M phosphate buffer; pH 7.4; 4°C) for morphological identification of the neurons. Tris buffered saline (in mM, Tris base, 8; Trisma HCl, 42; NaCl, 150; pH 7.4) supplemented with 0.1% Triton X-100 and 10% bovine serum (60 min) was used for permeabilization. Incubation was performed in phosphate buffer containing streptavidin-conjugated Alexa488 (1:300; Molecular Probes Inc., Eugene, OR, USA) for 90 min. The cells were visualized using a Zeiss LSM 510 confocal microscope (Carl Zeiss AG, Oberkochen, Germany).

Immunohistochemical experiments were carried out on 2 GFAP-tdTomato mice. Animals were transcardially perfused with Tyrode’s solution and a fixative (4% paraformaldehyde in 0.1 M phosphate buffer; PB, pH 7.4). After the transcardial fixation, the mesencephalon was removed and postfixed in the original fixative for 4 h, and 60 μm thick coronal slices were prepared with vibratome. Free-floating slices were first incubated either with rabbit anti-NeuN or guinea pig anti-GFAP antibodies (diluted 1:1000; Millipore and Synaptic Sytems, respectively) for 48 h at 4°C, and then anti-rabbit or anti-guinea pig IgG conjugated with Alexa Fluor 488 (diluted 1:1000; Molecular Probes Inc., Eugene, OR, USA) was applied on them.

Prior to the antibody application, the sections were incubated in 10% normal goat serum (Vector Labs, Burlingame, CA, USA) for 50 min. Antibodies were diluted in 10 mM Tris-buffered saline (TBS; pH 7.4) with 1% goat serum (Vector Labs, Burlingame, CA, USA).

### Reconstruction of Glial Elements and Neurons

After performing electrophysiological experiments and labeling, the visualization of the neurons was achieved by using confocal microscope (Zeiss LSM 510; Carl Zeiss AG, Oberkochen, Germany); images were taken with 20–40× objective and 1 μm *z* stacks. The reconstruction of neurons and astrocytes was performed with NeuroLucida software (MBF Bioscience, Williston, VT, USA); the distance of astrocytic and neuronal somata was determined by using the Imaris and the LSM Image Browser softwares. First, the number of astrocytes within 70 μm from the automatically defined center of the neuronal soma was determined by automated detection of the center points astrocytic somata with Imaris software (Bitplane AG, Zurich, Switzerland). Distances between the center of an astrocytic soma and the border of the closest neuronal soma, facing the given astrocyte, were measured manually, by using the ruler tool of the LSM browser software. The above mentioned morphometric data were correlated with the frequency and area of the SICs recorded from neurons.

All data represent mean ± SEM. Student’s *t*-test was applied for assessing statistical significance (level of significance = *p* < 0.05). Pearson’s correlation coefficient (*r*) was applied as the measure of linear correlation between two independent datasets.

## Results

### SICs Exist on a Population of PPN Neurons

First, we investigated the presence of SICs on PPN neurons. Fifteen of them (from seven mice) were patch-clamped in nACSF with 1 mM Mg^2+^, where 40% of the neurons had slow events with comparable parameters to literature data (see Pál, 2015). The average frequency of these events was 0.09 ± 0.05/min, their amplitude was 43.3 ± 10 pA, with a rise time of 220.1 ± 33.1 ms and a decay tau of 706 ± 161 ms (fit with a single exponential function). The average area of these events was 25.4 ± 13.1 pC.

When nominally magnesium-free aCSF was used, 55.8% of them displayed SICs (*n* = 52 from 20 mice). The average frequency was 0.23 ± 0.06/min, with amplitude of 95.5 ± 7.2 pA, a rise time of 88.7 ± 8.4 ms, a decay tau of 285.3 ± 24.7 ms and an area of 33.7 ± 4 pC. Compared to the data recorded in nACSF, the amplitude of the events increased significantly (*p* = 0.01).

**Figure 1:**
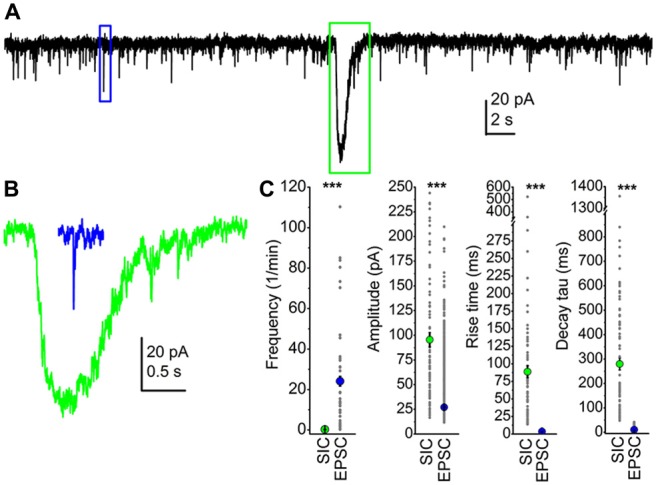
**Slow inward currents (SICs) can be unambiguously separated from excitatory postsynaptic currents (EPSCs). (A)** A representative trace of spontaneous events recorded from a pedunculopontine nucleus (PPN) neuron in voltage-clamp mode, at −60 mV holding potential. The blue square indicates a spontaneous EPSC (sEPSC), and a SIC is framed by the green square. **(B)** The representative sEPSC (blue) and SIC (green) from panel **(A)**, with the same scale. **(C)** Statistical summary of different parameters of SICs and sEPSCs. Green dots indicate the average ± SEM of the SIC parameters, whereas blue dots are the average ± SEM of sEPSC data. All differences are statistically significant (***p < 0.001). Gray dots represent individual data.

We compared parameters of SICs and EPSCs from the PPN in order to find unambiguous differences between them. First, the frequency of sEPSCs was two magnitudes higher: sEPSCs had a frequency of 25.6 ± 4.3/min, and the amplitude of the sEPSCs was 27.1 ± 0.16 pA, which was significantly different from the average SIC frequency and amplitude (*p* < 0.001). However, overlaps between individual amplitude values were seen, as sEPSC amplitudes were ranged between 11 and 209 pA, whereas SIC amplitudes were between 16.6 and 324 pA. Rise time of sEPSCs was 3.4 ± 0.02 ms, and declining phase of sEPSCs was correctly fit with a double exponential function, resulting τ_1_ of 4.3 ± 0.07 ms and τ_2_ of 43.7 ± 1.41 ms. In order to make the decay tau statistically comparable with data obtained from SICs, a single exponential fit of the declining phase of sEPSCs was performed, which resulted in 9.47 ± 0.2 ms as decay tau. Neither rise time nor decay tau of sEPSCs and SICs had a numerical overlap: rise time of sEPSCs was ranged between 0.7 and 7.9 ms, whereas it was between 13.8 and 522 ms for SICs. Similarly, decay tau of sEPSCs obtained with a single exponential fit was between 1.2 and 41.8 ms, but for SICs it was ranged between 48 and 1356 ms. The average area of sEPSCs was 223.8 ± 2.4 fC, which was in strong contrast with the SIC area of 33.7 ± 4 pC. Differences of sEPSC and SIC amplitude, rise time, decay tau and area were statistically significant (*p* < 0.001; Figure 1). Although great differences were seen in the frequency and the area of SICs and sEPSCs, the total charge movement per unit time by the two types of events were comparable (4.2 ± 0.8 pC/min for sEPSCs and 11.4 ± 7.5 pC/min for SICs; *p* = 0.12).

Based on our results, we can conclude that SICs exist on PPN neurons, and the two types of excitatory events (SICs and sEPSCs) can be clearly distinguishable according to rise time and decay tau.

### Astrocytic Glutamate Release and GluN2B Subunit Containing NMDA Receptor Activation Is Responsible for SICs

When 1 μM TTX was applied in a magnesium-free aCSF, 57.1% of the neurons displayed SICs (*n* = 14 from seven mice). The average frequency of these events was 0.21 ± 0.12/min, with amplitude of 57.9 ± 5.7 pA, rise time of 103.4 ± 12.8 ms, decay tau of 428.4 ± 41.7 pA and area of 22.2 ± 3 pC. Compared to the recordings in magnesium-free control solution, the amplitude was significantly decreased (*p* = 0.004) and a significant increase of decay tau was seen (*p* = 0.002).

**Figure 2:**
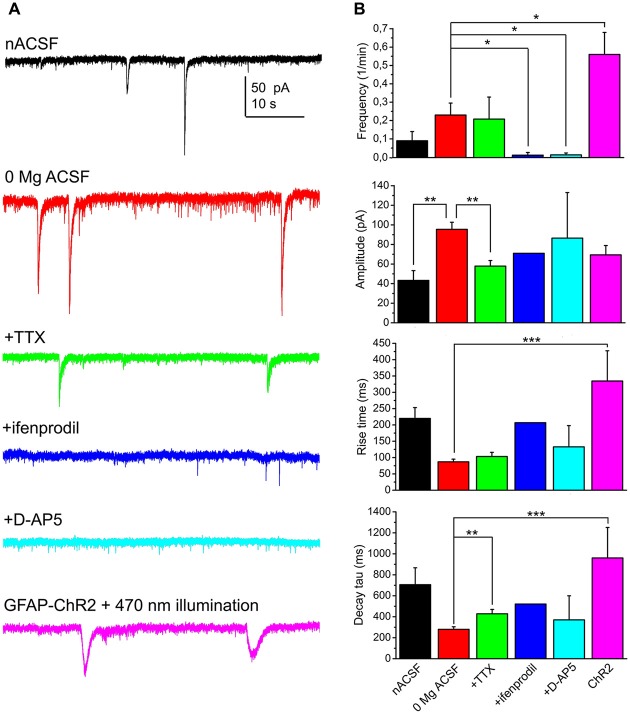
**SICs of the PPN neurons are consequences of NMDA receptor activation and astrocytic activity. (A)** Representative voltage clamp traces from PPN neurons under different conditions (black: normal artificial cerebrospinal fluid [nACSF], *n* = 15; red: magnesium-free ACSF, *n* = 52; green: magnesium-free ACSF + tetrodotoxin (TTX), *n* = 14; blue: magnesium-free ACSF + ifenprodil [GluN2B subunit-specific NMDA receptor inhibitor], *n* = 12; light blue: magnesium-free ACSF + D-AP5 [general NMDA receptor inhibitor], *n* = 22; magenta: magnesium-free ACSF; recorded from glial fibrillary acidic protein (GFAP)-channelrhodopsin-2 (ChR2) sample illuminated with 470 nm light). **(B)** Statistical comparison of SIC parameters using the same color code as on panel **(A)**. (*p < 0.05; **p < 0.01; ***p < 0.001).

In order to find the neuronal receptor responsible for these events, 50 μM D-AP5 (the general blocker of NMDA receptors) or 5 μM ifenprodil (GluN2B subunit specific NMDA receptor inhibitor) was used. After washing the preparation for 3 min with these drugs, SICs disappeared in the majority of the cases. However, a single SIC was seen when ifenprodil was applied (*n* = 12 from three mice; on 8.3% of all the neurons) and SICs were detected in two cases when D-AP5 was administered (*n* = 22 from nine mice; on 9.1% of the neurons; Figure 2).

In the next set of experiments we sought evidence for the relationship between astrocytic activation and neuronal SIC generation. First, the slices were treated with 1 μM thapsigargin for 45 min prior to recording. This drug, as a potent, but non-specific blocker of astrocytic activity, depletes intracellular calcium stores by blockade of sarco/endoplasmic reticulum Ca^2+^-ATPase and prevents generation of astrocytic slow calcium waves (Kirischuk et al., 1995; Nimmerjahn et al., 2004; Kőszeghy et al., 2015; see Verkhratsky et al., 2012). Under the same experimental arrangement described above, no SICs were detected from incubated slices (*n* = 8 from four mice). Second, mice expressing ChR2 in a GFAP-dependent way were used for optogenetic activation of astrocytes and observation of the consequential neuronal currents. Optogenetic activation of astrocytes and development of neuronal tonic currents were previously evaluated in our other study (Kovács et al., 2017). Briefly, slices from these mice were loaded with Oregon Green BAPTA I AM calcium indicator, and imaging was performed before, during and after the illumination of the slice with 470 nm light. Illumination elicited a significant increase of slow calcium waves. Whole-cell patch clamp experiments on cells with slow calcium waves revealed their non-excitable nature and the existence of cca. 65 pA photocurrent on them. Furthermore, specificity of the GFAP-cre line within the PPN is demonstrated by Figure 4.

Besides eliciting tonic neuronal currents, the frequency of SICs increased to 0.55 ± 0.11/min during the 1 min illumination of the sample with 470 nm light and in the next 2 min (when the majority of the astrocytes still had increased calcium wave frequency and neurons displayed tonic currents; Kovács et al., 2017). However, the kinetics of these events significantly differed from spontaneous SICs in their longer rise time and decay tau (334 ± 92 ms, 960 ± 291 ms, respectively; *p* < 0.001 in both cases), but not in amplitude (69.4 ± 9.5 pA; *n* = 15 from eight mice; Figure 2).

According to these experiments, astrocytic activity and GluN2B-containing NMDA receptor activation have a significant contribution to generation of SICs.

### Subpopulations of SIC-Possessing Neurons Are Determined by the Vicinity of Astrocytic Processes

**Figure 3:**
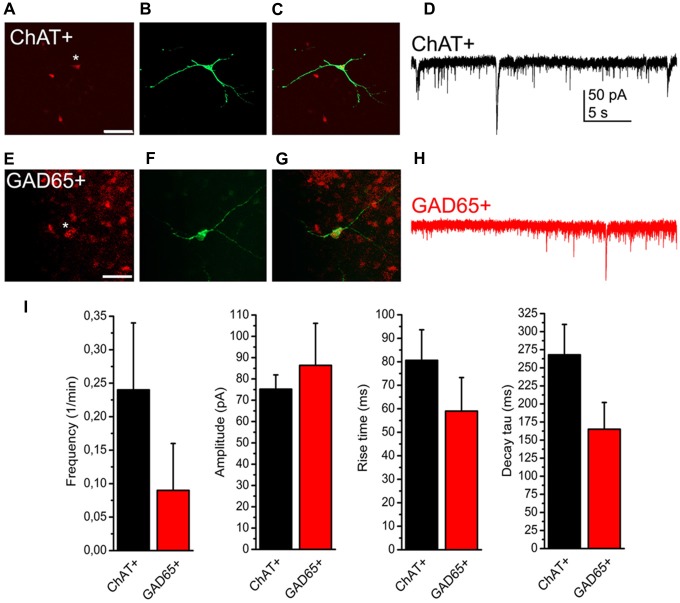
**Presence or absence of SICs is not related to neurochemical cell type. (A–C)** Identification of a cholinergic neuron. **(A)** tdTomato expression in a choline acetyltransferase (ChAT-) dependent way (red). The asterisk indicates the patched soma. Scale bar = 50 μm. **(B)** Biocytin labeling of the neuron indicated with asterisk on panel **(A**; green**)**. **(C)** Merged image of panels **(A,B). (D)** Voltage clamp recording from the neuron shown on panels **(A–C)**. Scale bar = 50 μm. **(E–G)** Identification of a GABAergic neuron. **(E)** Glutamate decarboxylase (GAD) 65-dependent tdTomato expression (red). **(F)** Biocytin labeling of the neuron indicated with asterisk on panel **(E**; green**)**. **(G)** Merged image of panels **(E,F). (H)** Voltage clamp trace from the neuron shown on panels **(E–G). (I)** Statistical summary of SIC parameters (black: cholinergic; red: GABAergic neuron; average ± SEM; *n* = 18 for ChAT- and *n* = 10 for GAD65-positive neurons). None of the differences are statistically significant.

As only slightly more than half of the neurons displayed SICs in our preliminary experiments, we hypothesized that the presence or absence of SICs might determine functional subtypes of PPN neurons. As the PPN is formed by distinct neurochemical subgroups of neurons (i.e., cholinergic and non-cholinergic -GABAergic and glutamatergic-neurons), experiments were conducted on genetically identified PPN neurons expressing tdTomato fluorescent protein in a ChAT- or GAD65-dependent way. Comparing data of SICs recorded on these neuronal subpopulations, no significant difference was found in frequency, amplitude, rise time and decay tau of these events (*p* = 0.14; *n* = 18 for ChAT- and *n* = 10 for GAD65-positive neurons; eight and seven mice, respectively; Figure 3).

**Figure 4:**
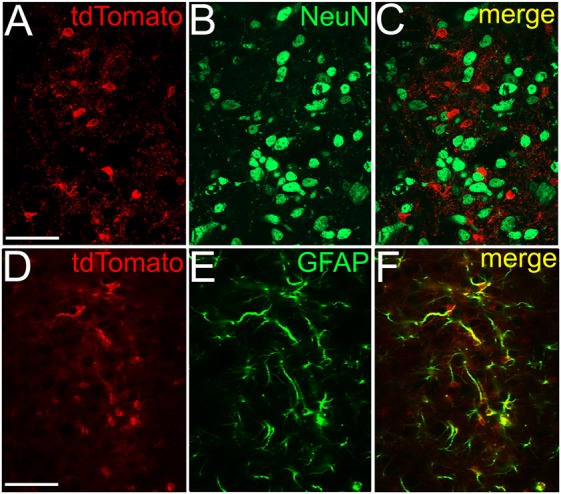
**NeuN immunopositivity never, GFAP immunopositivity strongly overlaps with GFAP-dependent tdTomato expression. (A)** GFAP-dependent tdTomato expression in the PPN. **(B)** NeuN immunopositivity in the region of the same area. **(C)** Merged image. **(D–F)** Colocalization of GFAP-dependent tdTomato expression **(D)** and GFAP-immunopositivity **(E)** with the same arrangement as on panels **(A–C)**. All images are single 1 μm thick *Z* stacks. Scale bar = 50 μm.

Next, we attempted to observe the dependence of SIC amplitude and area from the astrocyte-neurons connections. To achieve this, mice expressing tdTomato in a GFAP-dependent way was used. For validation of the GFAP-dependent tdTomato expression, immunohistochemical labeling of the neuronal marker NeuN and the astrocytic marker GFAP was performed on GFAP-tdTomato mice and colocalization of immunopositive somata or processes and genetically labeled somata and processes was quantified. Evaluation of the immunohistochemical labeling of the neuronal marker NeuN revealed that none of the somata possessed tdTomato expression in the area of the PPN (219 somata from six slices of two mice; Figures 4A–C). After GFAP-immunolabeling, 90 GFAP-tdTomato somata were counted from six slices of two mice, in the region of the PPN. Seventy-nine of them showed overlap or connection with GFAP-immunopositive structures (88.7 ± 3.3%; Figures 4D–F).

**Figure 5:**
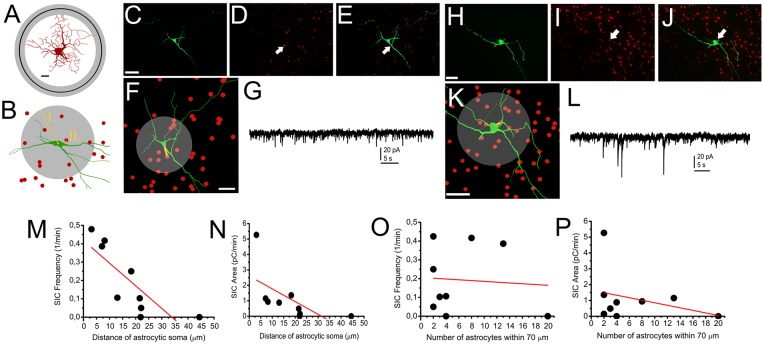
**Occurrence of SICs is determined by astrocytes being in close contact with neuronal somata. (A)** A reconstructed astrocyte with circles indicating the average distance of the farthest astrocytic end feet from the middle of the astrocytic soma (black circle: average; gray circle: SEM; scale bar = 10 μm). **(B)** Schematic drawing of morphological analyses resulting the data used for statistics. I., gray circle: astrocytic count was determined within 70 μm from the middle of the astrocytic soma. II. The shortest distance of the closest astrocyte and the neuronal soma. **(C–G)** and **(H–L)** comparison of SIC recordings and astrocytic position to neuronal somata demonstrated with two representative cases. **(C)** Biocytin labeling of a neuron. Scale bar = 50 μm. **(D)** tdTomato fluorescent protein expressed in a GFAP-dependent way. **(E)** Merged image of **(C,D)** panels. The arrows on **(D,E)** indicate the position of the nearest astrocytic soma to the neuronal soma. **(F)** NeuroLucida reconstruction of the neuron (green) and the positions of astrocytic somata (red). The yellow line indicates the distance between the nearest astrocytic soma and the neuronal soma, whereas the gray circle indicates the area for determining astrocyte count. Scale bar = 50 μm. **(G)** Voltage clamp recording from the neuron on panels **(C–F). (H–L)** Another neuron on panels with similar arrangement like **(C–G). (M–P)** Statistical summary of experiments depicted above. **(M)** Frequency of SICs plotted against the manually measured distance between the neuronal and the nearest astrocytic soma. **(N)** Area of SICs plotted against the manually measured distance between the neuronal and the nearest astrocytic soma. **(O)** Frequency of SICs plotted against the number of astrocytes within 70 μm from the middle of neuronal soma. **(P)** Area of SICs plotted against the number of astrocytes within 70 μm from the middle of neuronal soma. Black dots: individual data, red line: linear fit of the dataset (*n* = 9).

Next, neurons of GFAP-tdTomato mice were patched, labeled with biocytin and reconstructed. Distances between the middle of astrocytic somata and the closest point of neuronal soma, as well as the number of astrocytes within 70 μm from the mid point of neuronal soma were determined (as the average longest diameter of neuronal somata was 24.1 ± 2.1 μm and the farthest astrocytic end foot was 54.1 ± 6.6 μm away from the middle of astrocytic soma; Figures 5A–L). We found that linear correlation exists between the frequency and area of SICs recorded from a neuron and the distance of the closest astrocytic soma: the closer an astrocyte was located to the soma, the larger area or higher frequency of SICs was seen (*r* = −0.803 for SIC frequency and astrocyte-neuron distance; Figure 5M; *r* = −0.621 for SIC frequency and astrocyte-neuron distance; Figure 5N). When the number of astrocytes within 70 μm from the middle of the neuronal soma was automatically determined, *p* values did not indicate the existence of linear correlation (*r* = −0.074 for SIC frequency and the number of astrocytes within 70 μm; Figure 5O; *r* = −0.306 for SIC area and the number of astrocytes within 70 μm; *n* = 10 from nine mice; Figure 5P).

We concluded that differences in the occurrence of SICs are largely due to the anatomical situation of the neuronal soma and a single astrocyte being in close contact with the soma; instead of the neurochemical cell type of the neuron or the number of astrocytic somata being within a close distance from the neuronal soma.

### SICs Can Elicit Action Potential Firing on PPN Neurons but Do Not Synchronize Them

**Figure 6:**
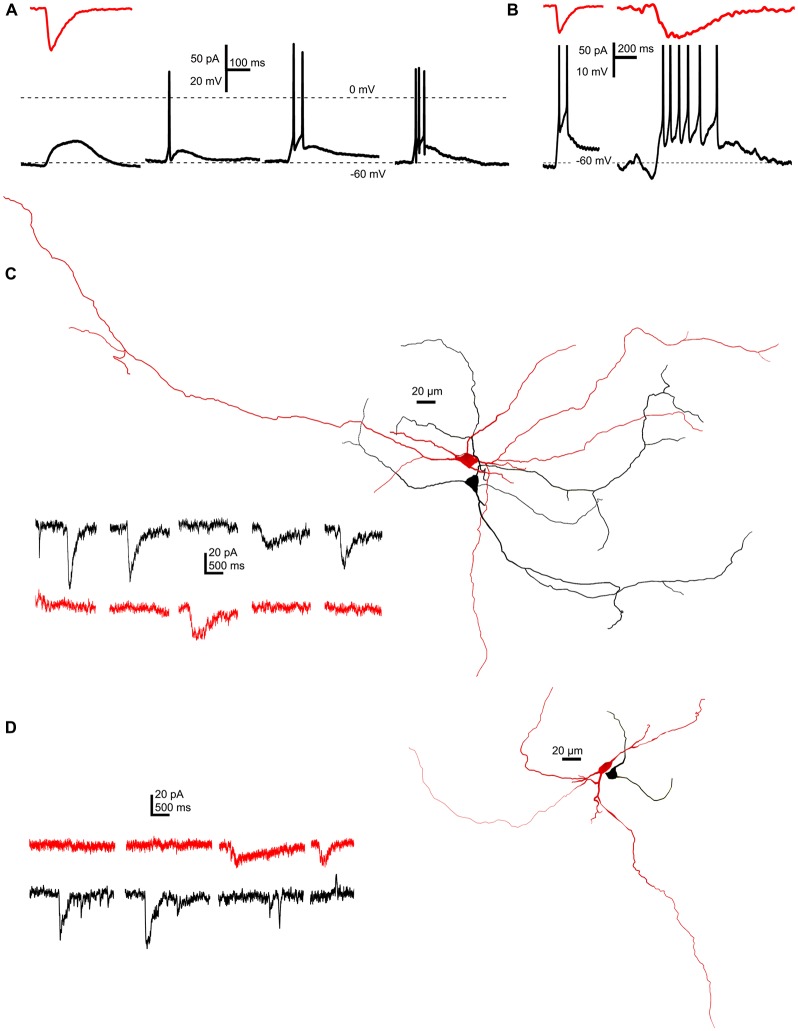
**SICs are capable of eliciting single action potentials or short action potential trains, but do not appear synchronously on neighboring neurons. (A)** A representative SIC (red trace, above) used as input signal in current clamp mode elicited electrotonic depolarization (12.5% of all cases), a single action potential (65%) or short trains consisting two or three action potentials (15 and 7.5%, respectively) on different PPN neurons held at −60 mV resting membrane potential (*n* = 40). **(B)** Using different SICs (red traces, above) shorter or longer trains of action potentials could be elicited on the same neuron. **(C,D)** Two examples of simultaneous recordings of synaptically non-coupled PPN neurons with somata within 50 μm from each other. Representative current traces from which parts without SICs were hidden (right). Red traces were recorded from the red, black traces were recorded from the black neuron from the corresponding reconstructed image on the left side (*n* = 9).

In the next series of experiments, the functional significance of SICs was sought. A representative SIC (89 pA amplitude, 18.8 ms rise time, 80 ms decay tau) was used as an input signal, when PPN neurons were patch-clamped in current-clamp configuration. Starting from −60 mV resting membrane potential, depolarization elicited by the injected current signal failed to fire neurons in 12.5% of all cases, a single spike was fired in 65%, and a short train of two or three action potentials was fired by 15 or 7.5% of the neurons (Figure 6A). When a larger and longer SIC was injected (102 pA amplitude, 134 ms rise time, 346.1 ms decay tau), longer trains of 5–8 action potentials were fired (*n* = 40 from 17 mice; Figure 6B).

Next, we sought evidence whether SICs can potentially synchronize firing of neighboring neurons. Pairs of synaptically non-coupled neurons were patched where somata were within 20 μm, and spontaneous activity was recorded in parallel in voltage clamp mode, at a holding potential of −60 mV. Thirty-two SICs were recorded from nine pairs, but none of the recorded events appear at the same time on the neighboring neurons (Figures 6C,D).

To conclude, SICs have a relatively high chance to elicit action potential firing on neurons but they do not seem to synchronize neighboring neurons.

### SICs Participate in Neuromodulatory Actions

We previously showed that endocannabinoids are capable of activating astrocytes in the PPN, which, in turn, elicit tonic currents on neurons in a metabotropic glutamate receptor dependent way (Kőszeghy et al., 2015; Kovács et al., 2017). Together with tonic currents, SICs also occurred (Kovács et al., 2017). In the next series of experiments, the relationship of different neuromodulatory agents, tonic currents and SICs were investigated. Holding currents and spontaneously developing SICs were recorded at a holding potential of −60 mV in voltage clamp mode, and different neuromodulatory agents (as the CB1 receptor agonist WIN55,212-2, 1 μM; the muscarinic agonist carbachol, 50 μM; serotonin, 10 μM) were applied in nominally magnesium-free solutions.

**Figure 7:**
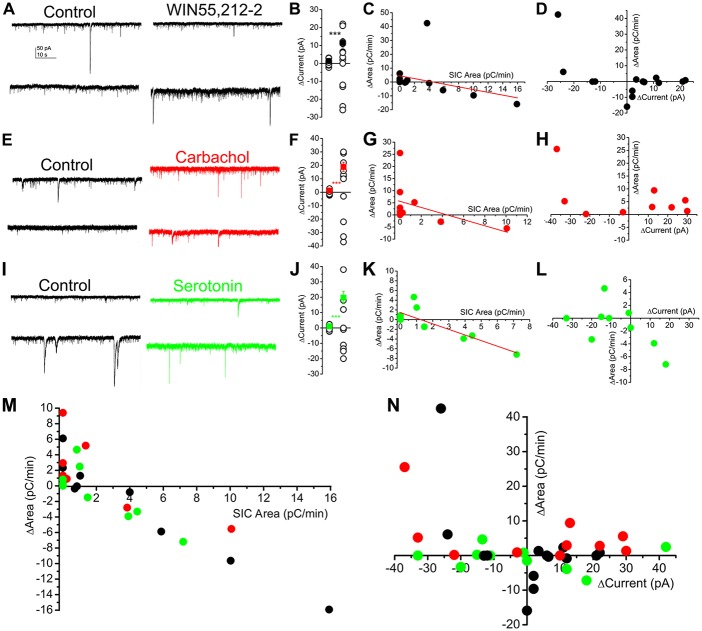
**SICs are uniformly involved in several neuromodulatory actions and represent a mechanism largely independent from tonic currents. (A–D)** Actions of WIN55,212-2 (CB1 receptor agonist; 1 μM) on SICs and tonic currents of PPN neurons. **(A)** Two representative voltage clamp traces under control conditions in nominally magnesium-free ACSF) and in the presence of WIN55,212-2. **(B)** Statistical summary of changes in the tonic current elicited by WIN55,212-2. The first dataset of hollow circles represents the spontaneous fluctuation of the baseline, whereas the second dataset shows changes elicited by WIN55,212-2. Black dots represent the average ± SEM of the absolute values of the two datasets (***p < 0.001). **(C)** Changes of SIC areas in the presence of WIN55,212-2 (“ΔArea”) plotted against SIC areas under control conditions (“SIC Area”). Black dots represent individual data; the red line is the result of the linear fit. **(D)** Changes of the areas of SICs (“ΔArea”) in the presence of WIN55,212-2 plotted against tonic changes of the holding current (“ΔCurrent”; *n* = 14). **(E–H)** Actions of the muscarinic agonist carbachol (50 μM) on SICs and tonic currents of PPN neurons with a similar arrangement as on panels **(A–D**; *n* = 11; ***p < 0.001). **(I–L)** Effects of serotonin (10 μM) on SICs and tonic currents of PPN neurons (*n* = 12); ***p < 0.001. **(M,N)** Summary of all investigated neuromodulatory actions on SICs and tonic currents. **(M)** Changes of SIC areas in the presence of neuromodulatory drugs (“ΔArea”) plotted against SIC areas under control conditions (“SIC Area”). **(N)** Changes of the areas of SICs (“ΔArea”) in the presence of neuromodulatory drugs (black: WIN55,212-2, red: carbachol; green: serotonin) plotted against tonic changes of the holding current (“ΔCurrent”).

When the CB1 receptor agonist WIN55,212-2 was used, seven neurons displayed tonic outward currents, three did not respond (the lack of response was defined as a change of tonic current within 3.5 pA; Kovács et al., 2017), and four neurons had tonic inward currents. The average of the absolute value of change (11.5 ± 2.34 pA) significantly exceeded the spontaneous fluctuation of the baseline current (1.34 ± 0.23 pA; *p* < 0.001; *n* = 14 from five mice; Figures 7A,B). Changes of the area of SICs seemed to have a weak linear dependence on the SIC area measured under control conditions (*r* = −0.36; Figure 7C). Below 3 pC/min area of SICs under control conditions, CB1 receptor stimulation was proved to increase SIC area, whereas the same action inhibited SICs above the aforementioned control value. In the next step, changes of the tonic currents and SIC amplitude were analyzed. In case of an existing correlation, one would hypothesize that the development of both inward and outward tonic currents are coupled with the increase of SIC area, as it was previously shown that inward and outward currents are due to activation of different sets of mGluRs (Kőszeghy et al., 2015; Kovács et al., 2017), thus tonic currents are consequences of glutamate release, which can potentially activate NMDA receptors in a transient way, as well. When absolute changes of the tonic currents and changes of SIC area were correlated, a weaker, but potentially existing relationship seemed to be likely (*r* = 0.45; Figure 7D).

Application of the muscarinic agonist carbachol also elicited tonic currents and affected SICs in a dual way. Seven of the neurons displayed tonic outward, whereas three of them had tonic inward currents, and a single neuron failed to respond to application of carbachol (*n* = 11 from four mice; Figures 7E,F). The absolute change of the holding current by carbachol was 18.9 ± 3.42 pA (the spontaneous fluctuation of the holding current was 1.24 ± 0.25 pA; *p* < 0.001). Similar to WIN55,212-2, if the area of SICs was less than 3 pC/min, carbachol elicited increase of this parameter in certain cases, whereas the same drug decreased SIC area if this parameter was originally higher than 3 pC/min. The linear fit of the dataset revealed only a weak correlation between the original SIC area and the change of this parameter by carbachol (*r* = −0.46; Figure 7G). Correlating absolute changes of tonic currents and changes of SIC area elicited by carbachol revealed that the possibility of the linear relationship between these parameters is low (*r* = 0.32; Figure 7H).

With serotonin, four of the investigated neurons had tonic outward and six of the neurons displayed tonic inward currents, whereas two of the neurons did not respond (*n* = 12 from five mice; Figures 7I,J). The absolute value of changes in tonic current was 19.72 ± 4 pA, which significantly differed from the spontaneous fluctuations (1.1 ± 0.3 pA; *p* < 0.001). Increase of the SIC area was characteristic for those neurons which had higher SIC area than 3 pC/min, and SIC generation was inhibited above that control area value. Fitting the dataset revealed a clear inverse proportionality between the original value and changes of SIC area by serotonin (*r* = −0.997; Figure 7K). When absolute changes of tonic currents and SICs were compared in a similar way like above, weak negative correlation was found (*r* = −0.305; Figure 7L).

Taken together, although in different extent, neuromodulatory agents act on SIC generation or inhibition, in a way depending on the starting value of SIC activity. Modulation of SIC activity with sporadic exceptions in endocannabinoid and cholinergic systems—seems to work as a system partially independent from generation of tonic currents (Figures 7M,N).

### SICs Are Likely Affected by NMDA Receptor Inactivation/Desensitization

**Figure 8:**
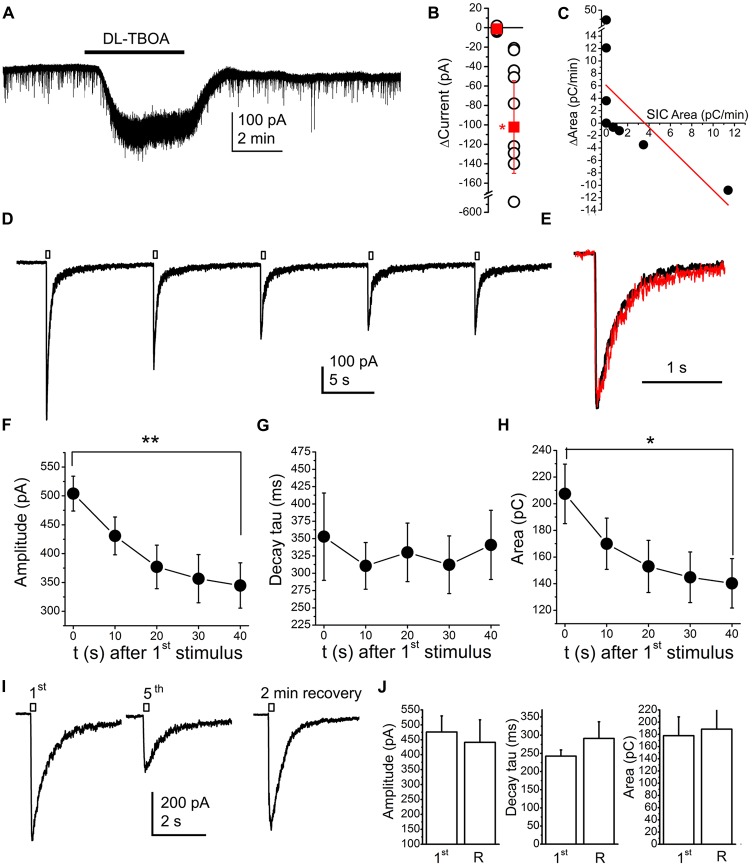
**Application of the glutamate transporter inhibitor DL-threo-β-Benzyloxyaspartic acid (DL-TBOA; 100 μM) or glutamate uncaging (100 μM MNI-caged-glutamate) models accumulating neuromodulatory actions. (A)** A representative trace of DL-TBOA application. **(B)** Statistical summary of inward currents elicited by TBOA (first dataset: spontaneous fluctuations of the holding current, second dataset: changes elicited by TBOA; hollow circles: individual data; red squares: average ± SEM; *p < 0.05) **(C)** Changes of the area with TBOA (“ΔArea”) plotted against area of SICs under control conditions (“SIC Area”; black dots: individual data, red line: linear fit; *n* = 9). **(D)** Result of repetitive glutamate uncaging in the presence of the AMPA receptor inhibitor 2,3-dihydroxy-6-nitro-7-sulfamoyl-benzo[f]quinoxaline-2,3-dione (NBQX; 10 μM), on a voltage clamp record. **(E)** The first (black) and fifth current (red) elicited by glutamate uncaging after amplitude alignment. **(F–H)** Statistical summary of changes of amplitude **(F)**, decay tau **(G)** and area **(H)** of currents elicited by repetitive glutamate uncaging (*n* = 10; *p < 0.05; **p < 0.01). **(I)** Representative current traces of the currents elicited by the first and fifth glutamate uncaging, and the current trace after stopped perfusion and 2 min of recovery. **(J)** Statistical summary of the amplitude, decay tau and area of currents elicited by glutamate uncaging at the first time (“1st”) and after a train of uncaging and 2 min of recovery with stopped perfusion (“R”; *n* = 5).

Actions of neuromodulatory agents on SICs depending on baseline activity might be consequences of NMDA receptor inactivation or desensitization by glutamate accumulation. In order to investigate this possibility, we artificially increased glutamate concentration in the vicinity of the investigated neuron by using the glutamate uptake inhibitor DL-TBOA (100 nM; Shimamoto et al., 1998; Jabaudon et al., 1999). Application of TBOA resulted an inward current of −102.25 ± 47.66 pA (the spontaneous fluctuation of the holding current was −1.21 ± 0.63 pA; *n* = 9 from seven mice; *p* = 0.011; Figures 8A,B). As expected, together with the occurrence of the tonic inward current, changes of SICs were also seen. If the original area of SICs was lower than 3 pC/min, increase of the area took place in certain cases and inhibition did not occur in another cases; whereas TBOA decreased the area of SICs in those cases where the SIC area was originally higher. Similar to the observations with neuromodulatory agents, an inverse linear correlation was observed between the original SIC activity and its changes during drug application (*r* = −0.59; Figure 8C). When occurrence of the tonic inward current and changes of SICs were compared, no correlation was found between the two phenomena (*r* = −0.005; data not shown).

Results with TBOA raised the possibility that inhibition of SICs was due to inactivation or desensitization of NMDA receptors. To elaborate on this hypothesis, 100 μM MNI-caged glutamate was applied along with 10 μM NBQX (AMPA receptor inhibitor). When uncaging was performed in parallel with voltage clamp recording, a transient inward current developed (Figure 8D; *n* = 10 from three mice). When flash illumination was repeated with a frequency of 0.1 Hz during continuous perfusion, the amplitude of the inward current gradually decreased, and, after three or four pulses, it reached a steady state value (*n* = 5). The decay tau of the declining phase did not change, only the amplitude and the area of SICs decreased (Figures 8E–H).

In order to exclude the possibility of consumption of available caged molecules, the perfusion was stopped after the fifth flash and, after 2 min break of uncaging and perfusion, flash illumination was applied again. After the break, recovery developed; indicating that caged compound was available in the solution in an excess amount (*n* = 6; Figures 8I,J).

After this series of experiments, one might conclude that repeated application of glutamate possibly exerts its action by inactivation of neuronal NMDA receptors.

## Discussion

Summarizing our findings, SICs exist on the PPN neurons. Based on their slower kinetics, SICs can be unambiguously distinguished from synaptic events. In accordance with previous findings from other brain areas, they are elicited in an astrocyte- and (mostly GluN2B-containing) NMDA receptor-dependent way on neurons (Angulo et al., 2004; Fellin et al., 2004; Kozlov et al., 2006; D’Ascenzo et al., 2007; Nie et al., 2010; Reyes-Haro et al., 2010; Pirttimaki et al., 2011; Pirttimaki and Parri, 2012; see Pál, 2015). SICs of the PPN neurons can be measured on those neurons whose somata are located close to a nearby astrocyte. These events can fire most neurons of the PPN, but do not appear synchronously on the neighboring neurons.

SICs are similarly affected by endocannabinoid, cholinergic and serotonergic neuromodulatory mechanisms, as the SIC area is increased by these actions in case if it is low under control conditions; but if the area of SICs is higher under control conditions, neuromodulatory mechanisms lower it. The activity-dependent nature of neuromodulatory actions on SICs seems to be determined by the amount of released extrasynaptic glutamate and the rate of NMDA receptor inactivation.

### SICs as General Phenomena of the Central Nervous System

Presence of SICs is likely a general feature of the CNS, as it was described in the visual cortex (Chen et al., 2012; Perea et al., 2014), the hippocampus (Angulo et al., 2004; Fellin et al., 2004; Perea and Araque, 2005; Carmignoto and Fellin, 2006; Kozlov et al., 2006; Lauderdale et al., 2015), the nucleus accumbens (D’Ascenzo et al., 2007), the thalamus (Parri et al., 2001), the olfactory bulb (Kozlov et al., 2006), the medial nucleus of the trapezoid body (Reyes-Haro et al., 2010) and the spinal cord (Bardoni et al., 2010; Nie et al., 2010). NMDA receptor-dependent large depolarizing episodes were also reported from the prefrontal cortex (Gao and Goldman-Rakic, 2006).

As a novel description of a structure possessing SICs, we report here that neurons of the PPN also display SICs. Similar to SICs recorded from other structures, these events can be distinguished from EPSCs due to their lower rise and decay times (Fellin et al., 2004; Shigetomi et al., 2008; Bardoni et al., 2010; Reyes-Haro et al., 2010; see Pál, 2015). Parameters of SICs and EPSCs recorded from the PPN fit well the ranges represented by the literature.

In accordance with the literature data, the vast majority of SICs in the PPN are consequences of GluN2B-containing NMDA receptor activation (Angulo et al., 2004; Fellin et al., 2004; Kozlov et al., 2006; D’Ascenzo et al., 2007; Nie et al., 2010; Reyes-Haro et al., 2010; Pirttimaki et al., 2011; Pirttimaki and Parri, 2012; Lauderdale et al., 2015). This receptor subtype is typical in extrasynaptic location (see e.g., Papouin and Oliet, 2014); therefore, along with the presence of SICs when synaptic vesicle release was blocked, one can conclude that SICs are nonsynaptic events in the whole CNS including the PPN (Lauderdale et al., 2015; see Pál, 2015).

It is widely accepted that SICs are generated by astrocytic glutamate release (Araque et al., 1998; Angulo et al., 2004; Bardoni et al., 2010; Fellin et al., 2004; D’Ascenzo et al., 2007; Pirttimaki et al., 2011; Chen et al., 2012; Perea et al., 2014). The astrocytic origin of SICs in the PPN was confirmed by our results. First, thapsigargin incubation prevented SIC generation. As thapsigargin does not specifically block astrocytes, but critically affects their activity (Kirischuk et al., 1995; Kőszeghy et al., 2015), further confirmation of astrocytic involvement was needed. Selective activation of astrocytes with optogenetic methods significantly increased the amplitude of SICs. Interestingly, SICs elicited by optogenetic activation had significantly slower rise and decay times than the spontaneously occurring events. This phenomenon might be explained with the possible swelling of astrocytes during optogenetic activation. As opening of the ChR2 results influx of Na^+^, Ca^2+^ and H^+^, it might result influx of water and swelling-dependent gliotransmitter release via VRAC channels (Abdullaev et al., 2006; Kozlov et al., 2006; Gourine et al., 2010; Figueiredo et al., 2011; Beppu et al., 2014; Perea et al., 2014; Ji and Wang, 2015). Similarly slower SICs were recorded in hypoosmotic solution where the swelling of astrocytes was quantified. In parallel with astrocytic swelling, the volume of the extracellular space is reduced, and reduction of the diffusion rate of glutamate is possibly responsible for slower kinetics of SICs (Lauderdale et al., 2015).

As further evidence supporting the astrocytic origin of SICs in the PPN, we showed that both frequency and total charge movement by spontaneously occurring SICs are inversely proportional to the distance of astrocytic somata from the neuronal soma. This finding also explains the large differences of SIC frequency and area recorded from different neurons. It is very likely that the number of contacts between neurons and astrocytic endfeets are larger if the somata are closer to each other, thus the released glutamate might reach a higher concentration at the surface of certain neurons, leading to the appearance of the detectable SICs. Furthermore—as it can be learned from cases of reactive gliosis, where retraction or elongation of astrocytic processes can influence neuronal excitability (see Wang and Parpura, 2016)—diffusion properties or changes of the reuptake of glutamate can be also influenced by the density and anatomical position of astrocytic processes, which can have a relationship with the distance from astrocytic soma.

Surprisingly, application of TTX resulted SICs with smaller amplitude and longer decay tau, leaving the SIC frequency unaffected. These findings might also indicate that either network effects or persistent sodium current can modify parameters of these events (Takakusaki and Kitai, 1997).

It can be a matter of debate whether NMDA receptors involved in the astrocyte-dependent neuromodulatory actions are neuronal extrasynaptic or astrocytic receptors. It is well established that NMDA receptor subunits are expressed by astrocytes, mostly located on the astrocytic processes and form functional channels (Lalo et al., 2006; Verkhratsky and Kirchhoff, 2007; Castillo et al., 2013; Dzamba et al., 2013, 2015). However, region-specific differences were also reported: minimal or no NMDA current was found on astrocytes of the red nucleus and the cerebellum (Usowicz et al., 1989; Akopian et al., 1997), but functional NMDA receptors were present on cortical astrocytes (Dzamba et al., 2015). As an important difference between astrocytic and neuronal NMDA receptors, it was observed on astrocytes from different regions that their NMDA receptors are not under magnesium blockade, thus were potentially active at resting membrane potentials (Lalo et al., 2006; Castillo et al., 2013). Furthermore, in case of cortical astrocytes—which are known to possess GluN2B subunit-containing functional NMDA receptors—only a smaller fraction of astrocytes displays ifenprodil-sensitive NMDA-current (Lalo et al., 2006). As appearance of SICs was sensitive to the extracellular magnesium concentration and the blocking effect of ifenprodil was very similar to the inhibition caused by D-AP5, we can hypothesize that SICs of the PPN are very likely due to activation of neuronal and not astrocytic NMDA receptors. However, one can not clearly exclude the possibility that astrocytic NMDA receptors contribute to the generation of SICs.

### Specific Roles of SICs in the PPN

SICs seem to be involved in physiological or pathological synchronization of neighboring neurons. Simultaneously occurring SICs on neurons close to each other were shown in the ventrobasal thalamus, hippocampus and nucleus accumbens (Angulo et al., 2004; Fellin et al., 2004; D’Ascenzo et al., 2007; Pirttimaki et al., 2011). In contrast, such synchrony was largely absent in the medial nucleus of the trapezoid body (Reyes-Haro et al., 2010). In accordance with the latter paper, we failed to find coincidence in the occurrence of SICs on PPN neurons being close to each other; thus they possibly do not play a role in direct synchronization of PPN neurons close to each other. Our findings and literature data might suggest that neuronal synchronization by SICs is characteristic for rostral brain regions, whereas SICs of the brainstem have different functions.

When a previously recorded SIC was used as an input signal in current clamp, a single action potential or a short train of spikes was observed. Although this experimental arrangement faces with limitations (as using a uniform direct current injection instead of application of dynamic current clamp arrangement; e.g., Sharp et al., 1993; Jaeger and Bower, 1999), which might easily result overestimation of the role of these events in eliciting neuronal firing; one might conclude that a significant fraction of SICs can lead to neuronal action potential firing.

Glutamate released by astrocytes does not only generate SICs on neurons, but also elicits tonic excitatory currents. When the concentration of ambient glutamate was increased by blockade of glutamate transporters, the kinetics of SICs became slower (Fellin et al., 2004) and tonic inward currents appeared as well (Angulo et al., 2004), suggesting that the distance between the glutamate release site and the neuronal NMDA receptors affect the kinetics of NMDA dependent actions. We previously observed that both CB1 receptor agonists and optogenetic activation of astrocytes elicit tonic inward and outward currents together with SICs. Both excitatory and inhibitory tonic currents seemed to be consequences of neuronal and/or astrocytic mGluR activation, therefore we hypothesized that tonic glutamatergic actions are linked with increased SIC occurrence (Kőszeghy et al., 2015; Kovács et al., 2017).

Various neuromodulatory actions—as parts of sleep homeostasis—affect sleep and wakefulness via influencing the PPN. Intracerebroventricular administration or direct injection of cannabinoid agonists to the PPN increased REM sleep duration (Murillo-Rodríguez et al., 1998, 2001, 2008; Murillo-Rodríguez, 2008) or non-REM sleep duration (Bolla et al., 2008; Herrera-Solís et al., 2010). Injection of the muscarinic agonist carbachol to the PPN induced long-lasting REM-like states, as well as an increase in the amplitude and emergence probability of cortical gamma oscillations (Kinney et al., 1998; Valencia et al., 2014). Systemic injection of serotonin receptor agonists increased duration of the wake state along with reduction of slow wave and REM sleep duration; intra-PPN injection of the same agents also reduced REM sleep duration. Serotonin receptor agonists also triggered hippocampal theta activity (Bjorvatn and Ursin, 1998; Matulewicz et al., 2010).

In the cellular level, these various neuromodulatory actions elicit dual tonic actions on PPN neurons. Cholinergic stimulation resulted depolarization or hyperpolarization on PPN neurons, leaving a distinct neuronal population unaffected (Ye et al., 2010). Cannabinoid agonists exerted similar actions on the PPN, showing marked overlap with the cholinergic actions (Kőszeghy et al., 2015; Kovács et al., 2015, 2017). In overlap with carbachol-initiated hyperpolarization, serotonin also elicited tonic hyperpolarization on the same PPN neurons (Leonard and Llinás, 1994). In accordance with literature data and our previous findings, all types of tonic responses were seen with endocannabinoid, muscarinic and serotonergic actions. It is, however, a still open question whether these actions overlap because of a similarly acting astrocytic activation and whether differences between these actions are due to an additional neuronal component. We recently revealed the mixed astrocytic-neuronal actions of cannabinoids on the PPN (Kőszeghy et al., 2015; Kovács et al., 2017), and astrocytic activation by serotonergic or muscarinic stimulation and consequential actions on neurons are also known from different brain areas (Chen et al., 2012; Navarrete et al., 2012; see Hertz et al., 2015). However, addressing this question is the matter of further investigation.

In contrast with our expectations (i.e., area of SICs is increased proportionally to the occurrence of tonic currents), minimal correlation was revealed between SIC occurrence and development of tonic currents in most cases (except occasional cases with CB1 and muscarinic receptor agonists), suggesting that SICs and tonic currents usually occur separately in the PPN, representing two different astrocyte-dependent neuromodulatory systems with limited overlaps.

Also surprisingly, we found that changes in SIC frequency and area by neuromodulatory actions depend on previous SIC activity and not on the neuromodulatory agent itself: if original SIC activity was high, neuromodulatory actions decreased it, while in case of low SIC activity, neuromodulatory agents had a stimulatory action on them. Explaining these results along with the previously obtained data (Kőszeghy et al., 2015; Kovács et al., 2015, 2017), we hypothesize that neuromodulatory actions increase ambient glutamate level, which stimulate appearance of SICs by activating NMDA receptors, if the majority of NMDA receptors are available for activation. However, if NMDA receptors are already activated by a previously increased ambient glutamate level, further glutamate increase results NMDA receptor desensitization/inactivation. If NMDA (and likely mGluR) receptors were activated repeatedly, the amplitude of the elicited current decreased in a regenerative manner, but no changes of the declining phase were seen. In accordance with the literature (Vyklický, 1993), the background of this phenomenon is possibly the NMDA receptor inactivation.

### Functional Considerations

As the general physiological or pathophysiological roles of SICs are not always clearly understood, understanding the role of this phenomenon in the PPN might also have difficulties. It is generally thought that SICs are capable of synchronizing neighboring neurons (Angulo et al., 2004; Fellin et al., 2004; D’Ascenzo et al., 2007; Pirttimaki et al., 2011; see Pál, 2015), but this might be only the case in cortical and rostral subcortical brain areas (hippocampus, thalamus, nucleus accumbens). Brainstem SICs possibly have different functions, as neither our study nor a previous work (Reyes-Haro et al., 2010) identified abundant synchronization of these events on neighboring neurons.

It is a limitation of our study whether observation of SICs refers to physiological situation. SICs are usually recorded in magnesium-free solution which is known to provocate seizure-like activity in cortical preparations (e.g., Derchansky et al., 2004; Gao and Goldman-Rakic, 2006). The reason to use this solution is that the frequency of SICs is originally low, due to the magnesium blockade of NMDA receptors (Angulo et al., 2004; Fellin et al., 2004; D’Ascenzo et al., 2007). This might raise the possibility that SICs have limited physiological importance but crucial in some pathophysiological conditions e.g., stroke, epileptic seizures or cerebral edema (Wetherington et al., 2008; Dong et al., 2013; Lauderdale et al., 2015). Furthermore, coronal brainstem slices are disconnected from several inputs; thus our model only allows investigation of local networks and actions separated from most of their inputs.

The PPN is known as a regulator of sleep-wake cycles (Steriade et al., 1990, 1991; Garcia-Rill, 1991; Reese et al., 1995). In order to participate in its regulation, PPN neurons display firing patterns correlated with global brain states (see Saper et al., 2005; Mena-Segovia et al., 2008; Ros et al., 2010; Lee and Dan, 2012). PPN neurons show a high level of synchronization during cortical slow wave activity, but this population activity turns to be desynchronized during active state (Petzold et al., 2015). One might speculate that this desynchronization can be triggered or occur more effectively if certain neurons are forced to fire random spikes or short trains of action potentials, as it was observed when SICs were used as input signals for neurons. However, neuromodulatory actions on SICs seem to stabilize a certain level of desynchronization, as neuromodulatory agents (independently whether they are sleep- or wake-promoting) might increase or decrease SIC activity, depending on the original level of SICs and thus the desynchronization. Taken into account that neuromodulatory actions with different roles in sleep-wake regulation seem to have a uniform action on SICs, its suggested function in the PPN might be to provide a balanced level of desynchronization.

## Author Contributions

AK conducted and analyzed the experiments. BP designed, conducted, analyzed the experiments and wrote the article.

## Conflict of Interest Statement

The authors declare that the research was conducted in the absence of any commercial or financial relationships that could be construed as a potential conflict of interest.
